# Beyond words: understanding anxiety and depression in college applicants through LIWC analysis of textual features

**DOI:** 10.3389/fpsyg.2025.1690926

**Published:** 2025-12-04

**Authors:** Ana Abutara, Aline Kissimoto, Felipe Oliveira de Aguiar, Victor Otani, Ricardo Riyoiti Uchida, Lucas Murrins Marques

**Affiliations:** 1 Department of Mental Health, Santa Casa de São Paulo School of Medical Sciences, São Paulo, Brazil; 2 Division of Artificial Intelligence Research, Infinity Doctor’s Inc., DE, Miami, FL, United States

**Keywords:** exam, essay, LIWC, depression, anxiety, multivariate analysis

## Abstract

**Background:**

Anxiety and depression are highly prevalent among pre-university students, often intensified by the academic stress associated with entrance exams. Linguistic analysis of written texts offers a promising, non-invasive approach for early detection and prevention. Study design: Cross-sectional study.

**Objectives:**

To examine the association between linguistic features in essays and levels of anxiety and depression, identifying specific language patterns linked to these conditions.

**Setting:**

Pre-university preparatory courses in São Paulo, Brazil, in 2023.

**Methods:**

Participants were 62 pre-university students (51 females, 11 males; *M* = 20.3 years, SD = 2.65) who completed a self-report form shared via WhatsApp or in-person at preparatory schools. The form included sociodemographic questions, the GAD-7 and PHQ-9 scales, and the upload of an argumentative essay written within the previous month as part of their regular coursework. Essays were analyzed using LIWC software, and multivariate regression models identified linguistic features associated with anxiety and depression scores.

**Results:**

Higher anxiety levels correlated with increased use of words related to affiliation and home, and decreased use of leisure and money-related terms. Depression was associated with higher frequency of drives and number-related words, and fewer motion-related terms.

**Conclusion:**

Linguistic analysis can assist in identifying emotional distress among pre-university students, offering a potential tool for early screening and intervention in educational and mental health contexts.

## Introduction

Understanding anxiety and depression within the contemporary mental health landscape is crucial, especially during critical life stages such as adolescence and early adulthood, where external pressures profoundly impact daily life. These psychological disorders are not only prevalent but also have long-term detrimental effects on those affected. According to the World Health Organization (WHO), depression is the leading cause of disability worldwide, affecting more than 264 million people, while anxiety disorders affect more than 284 million people globally (World Health Organization [WHO], 2020). In this paper, we evaluate the anxiety and depression levels of students during their preparations for university entrance exams, a period characterized by intense academic and emotional stress.

Early identification of depressive and anxiety disorders in teenagers and young adults is critical for providing effective interventions and timely psychological support. The literature suggests that over 90% of suicide cases are associated with some form of mental disorder, with approximately 43.2% linked to major depression (de Barbosa et al., 2011). Early detection of depression and anxiety in youth could allow for prompt intervention, preventing future complications and the worsening of these cases. This could lead to reduced emotional suffering, improved overall functioning, and the promotion of healthy development. Moreover, addressing mental health issues early contributes to reducing stigma, encouraging more teenagers to seek help when needed, and fostering a supportive mental health culture.

The relationship between emotional states and language is well-documented in various studies. For example, research by Pennebaker and Seagal (2003) has demonstrated significant correlations between language characteristics and mental health states through the Linguistic Inquiry and Word Count (LIWC) tool, showing that higher levels of depression correlate with fewer expressions of emotion. Additionally, a study by Stirman and Pennebaker (2001) found that word usage patterns can predict psychological well-being, providing valuable insights into individuals’ mental states. Existing literature includes research on similar themes, such as a cross-sectional study analyzing Instagram posts of teenagers who committed suicide compared to those of living teens, identifying distinct linguistic patterns that could reveal underlying mental processes (Walker et al., 2024).

The LIWC application is a text analysis software that calculates the degree of use of different word categories in various texts (Pennebaker, 2001). This program uses a lexical resource known as the LIWC dictionary, which was made available in Portuguese in 2013 and updated in 2017 (Balage Filho et al., 2013). The LIWC operates based on two central features: the processing component and the dictionaries. The processing component is the program itself, which opens a series of text files—from essays to poems to novels—and scans each file word by word, comparing each word against the dictionary file. The program then evaluates the language based on psychological and social meanings, featuring 81 categorization classes. These linguistic categories encompass psychological dimensions such as emotional tone, cognitive processing, social orientation, and motivational drives. For example, the frequency of affective and cognitive words has been shown to reflect underlying emotional regulation and self-referential thinking, processes frequently altered in anxiety and depression.

In clinical contexts, Stirman and Pennebaker (2001) demonstrated that suicidal poets used more self-focused pronouns and fewer positive emotion words compared to non-suicidal ones, while Rude et al. (2004) found that depressed college students exhibited greater use of first-person singular pronouns and cognitive mechanism words, reflecting ruminative thinking. Similarly, Chung and Pennebaker (2011) showed that linguistic patterns can indicate emotional recovery and cognitive restructuring after stressful events. These findings suggest that LIWC captures subtle indicators of affective and cognitive states that are theoretically and empirically related to anxiety and depression.

The current literature shows a gap in studies that analyze the context of formal and evaluative texts written by students in pre-university periods. By identifying psychological processes in these essays, we can bring the following considerations to light: could mental disorders affect rigorously formal written texts? These exams, seemingly far from displaying students’ feelings and anguish, might be impaired by their mental suffering. A study published in 2013 demonstrated a significant association between increased depression and anxiety among students preparing for university entrance exams, recognizing the importance of seeking means to detect such cases early (Terra et al., 2013).

Given the importance of case identification in this population and the existing literature gap regarding studies that analyze language in formal texts, this study aims to explore how linguistic features in essays can predict levels of anxiety and depression among students preparing for university entrance exams. Through the LIWC and the generalized anxiety disorder 7-item (GAD-7) and Patient Health Questionnaire-9 (PHQ-9) scales, the diagnostic levels of anxiety and depression levels in the students will be compared with the linguistic characteristics identified in their essays through various LIWC categories. If the analysis of written language in young adults for the early detection of depressive and anxious disorders proves effective, it has the potential to improve the detection and treatment of depression in youth, contributing to a more holistic and personalized approach in psychiatric medical practice.

This research was divided into three stages. The first stage was data collection through an online form, including depression and anxiety scales, submission of essays, and sociodemographic data shared in WhatsApp groups and presented in person at pre-university courses. The second stage involved evaluating the essays using the LIWC software. The final stage included a multivariate linear regression analysis to examine the relationship between the variables (results from the PHQ-9 and GAD-7 scales and LIWC categories).

## Materials and methods

### Sample and structure

The Mental Health Department is a medical service dedicated to mental health education and practice in São Paulo, affiliated with the Santa Casa de São Paulo School of Medical Sciences.

For this study, data were collected using a form distributed via the WhatsApp application. The link was shared through groups of students attending pre-university courses and was also physically distributed in some courses in São Paulo (SP). The form included sections for submitting an essay written in the last month, as well as personal data such as age, gender, duration of attendance in the pre-university course, and the chosen university course. The research included students enrolled in pre-college courses during the year 2023 who were over 18 years old. Data collection was conducted from August to December 2023. The exclusion criterion for this study was students under 18 years of age.

This study was submitted to and approved by the Ethics Committee (CAAE: 53060321.8.0000.5479). The measurement instruments used, including the PHQ-9 and GAD-7 scales, are validated in Brazil. When necessary, cutoff values recommended by the validation studies were adopted. The following scales were employed:

### Sociodemographic questionnaire

In order to evaluate sociodemographic profile we exclusively assess the following variables: (i) Sex; (ii) Age; and (iii) number of years of preparatory course.

### Generalized anxiety disorder 7-item (GAD-7)

A brief screening tool used to detect symptoms of generalized anxiety disorder. It consists of seven questions that assess the frequency of core anxiety symptoms over the past 2 weeks. Responses are scored on a four-point Likert scale ranging from 0 (“Not at all”) to 3 (“Nearly every day”), with the total score ranging from 0 to 21. For this study we did not consider any threshold to identify potential cases of generalized anxiety disorder, only the total score as a continuous value.

### Patient Health Questionnaire-9 (PHQ-9)

A concise self-administered instrument for screening depression levels. The questionnaire includes nine items that inquire about the frequency of depressed mood and anhedonia over the past 2 weeks. Each item is scored from 0 (“Not at all”) to 3 (“Nearly every day”), leading to a total score that ranges from 0 to 27. The PHQ-9 helps in identifying individuals who may require a more comprehensive assessment for depression levels. As adopted for the GAD-7 scale, we did not consider any threshold to identify potential cases of depressive disorder, only the total score as a continuous value.

### Linguistic Inquiry and Word Count (LIWC) variables

In order to measure characteristics of the content of texts, essays were collected through the online form via WhatsApp in photo format. After the collection period, we transcribed the essays into online documents. The texts were analyzed using the Linguistic Inquiry and Word Count (LIWC) system, in version 22, seeking to extract 81 variables divided into 12 categories: (i) Summary variables - Word count, Big words, Dictionary words; (ii) Linguistic dimensions - Total function words, Total pronouns, Personal pronouns, We, You, She/He, They, Impersonal pronouns, Articles, Prepositions, Auxiliary verbs, Adverbs, Conjunctions, Negations, Common verbs, Common adjectives, Comparison words, Interrogatives, Number, Quantities; (iii) Drives - Affiliation, Achieve, Power; (iv) Cognitive Processes - Insight, Causation, Discrepancy, Tentative, Certitude, Differentiation; (v) Affect - Positive Emotion, Negative Emotion, Anxiety, Anger, Sadness; (vi) Social Processes - Family, Friends, Female references, Male references; (vii) Perception - Relativity, Motion, Space, Visual, Auditory, Feeling, Time, Past focus, Present focus, Future focus; (viii) Lifestyle - Work, Leisure, Home, Money, Religion, Death; (ix) Physical - Health, Sexual, Food, Death; (x) Motives - Reward, Risk; (xi) Conversational - Netspeak, Assent, Nonfluencies, Fillers; (xii) All punctuation - Periods, Comma, Question Mark, Exclamation points, Apostrophes, Other punctuation. These variables were selected to provide a comprehensive overview of textual characteristics that may be associated with mood variations, regardless of the theme of the text.

### Procedures

Individuals of both genders, aged 18 years or older, who were enrolled in pre-university preparatory courses in São Paulo were invited to participate. After providing digital informed consent, participants completed a self-report form distributed via WhatsApp or in-person at preparatory schools. The form included sociodemographic questions, the PHQ-9 and GAD-7 scales, and an upload field for a recent essay written within the previous month.

These essays were part of the students’ regular preparatory coursework for university entrance exams and followed the standard format adopted by most Brazilian preparatory schools — formal argumentative compositions of approximately 20–30 lines (around 200–300 words). The topics and purposes of these essays were not controlled by the researchers but were determined by the participants’ courses, ensuring that the texts reflected authentic linguistic production within a standardized academic context. This approach allowed us to capture naturally occurring written language under comparable formal and structural conditions.

After collecting the data and excluding incomplete responses, the essays were transcribed into digital text files for processing. The linguistic content was analyzed using the Linguistic Inquiry and Word Count (LIWC) software on personal computers, and the resulting output was organized into spreadsheets for subsequent statistical analysis.

### Data analysis

Statistical analysis was conducted using STATA 12.1^®^ (StataCorp LP, Texas). Scores for GAD-7 and PHQ-9 obtained from the included participants were used as dependent variables in univariate tests with all sociodemographic and LIWC variables as independent variables. Independent variables that (i) presented a *p*-value < 0.2 were included in the next step (multivariate regression) and (ii) those that did not reach significance were excluded using the backward stepwise regression method to avoid suppressor effects (Bursac et al., 2008).

Following this, we performed two multivariate linear regression analyses to separately measure associations between GAD-7 and PHQ-9 scores as dependent variables, and all variables included in the previous step for each dependent variable. As determined by the literature, all models tested four assumptions: linearity, homoscedasticity, independence, and normality (Osborne and Waters, 2002). These assumptions were evaluated through graphical inspection of residual plots and statistical diagnostics. Linearity and homoscedasticity were assessed via scatterplots of standardized residuals versus predicted values. Normality of residuals was verified through histograms, Q–Q plots, and the Kolmogorov–Smirnov test. Independence of residuals was examined using the Durbin–Watson statistic. To assess potential multicollinearity, we calculated Variance Inflation Factor (VIF) values for all predictors; all VIFs were below five, indicating no collinearity concerns. All assumptions were satisfactorily met for the final models.

Confounders such as age and sex were tested in all models. These confounders were selected prior to analysis based on discussions between the authors and existing literature. A variable was considered a confounder if it changed the β coefficient by more than 10%.

## Results

Considering the 62 participants, we had no exclusions during the analysis process, as the quality and frequency of the data were preserved. The final sample consisted of 62 college applicants (51 females, 11 males), aged between 18 and 25 years (*M* = 20.3, SD = 2.65). The majority were attending their first or second year of preparatory courses (*M* = 1.94 years, SD = 1.60). These characteristics are summarized in Table 1 below.

**TABLE 1:** Mood, sociodemographic and Linguistic Inquiry and Word Count (LIWC) variables.

Mood	Mean (Standard deviation)
PHQ-9	11.82 (±5.16)
GAD-7	11.06 (±5.16)
**Sociodemographic**	
Sex	Female (51)
	Male (11)
Age	20.3 (±2.65)
Years of preparatory course	1.94 (±1.60)
**Summary variables**	
Word count	360.53 (±71.41)
Big words	34.50 (±3.47)
Dictionary words	76.09 (±3.81)
**Linguistic dimensions**	
Total function words	50.95 (±2.59)
Total pronouns	19.88 (±2.82)
Personal pronouns	10.77 (±1.62)
We	0.13 (±0.27)
You	0.70 (±0.59)
She/he	8.45 (±1.66)
They	2.42 (±1.06)
Impersonal pronouns	16.90 (±2.46)
Articles	16.39 (±2.01)
Prepositions	22.89 (±2.43)
Auxiliary verbs	4.38 (±1.59)
Adverbs	9.71 (±2.35)
Conjunctions	9.54 (±2.07)
Negations	1.44 (±0.95)
Common verbs	8.88 (±2.17)
Common adjectives	4.56 (±1.73)
Comparison words	2.77 (±1.10)
Interrogatives	4.61 (±1.28)
Number	2.30 (±0.90)
Quantities	1.80 (±1.21)
**Drives**	9.75 (±2.88)
Affiliation	2.00 (±1.71)
Achieve	2.02 (±1.18)
Power	3.12 (±1.60)
**Cognitive processes**	13.09 (±2.60)
Insight	1.95 (±1.13)
Causation	4.16 (±1.18)
Discrepancy	2.10 (±0.85)
Tentative	1.96 (±0.95)
Certitude	1.39 (±0.69)
Differentiation	3.41 (±1.36)
**Affect**	4.57 (±1.92)
Positive emotion	2.28 (±1.32)
Anxiety	0.31 (±0.57)
Anger	0.57 (±0.71)
Sadness	0.29 (±0.31)
**Social processes**	5.89 (±2.48)
Family	0.40 (±1.07)
Friends	0.34 (±0.80)
Female references	0.45 (±0.80)
Male references	0.75 (±0.71)
**Perception**	1.87 (±1.11)
Relativity	10.83 (±2.63)
Motion	3.10 (±1.33)
Space	6.59 (±1.78)
Visual	1.10 (±0.84)
Auditory	0.36 (±0.60)
Feeling	0.54 (±0.49)
Time	4.30 (±1.91)
Past focus	1.13 (±1.19)
Present focus	6.63 (±1.88)
Future focus	0.31 (±0.29)
**Lifestyle**	
Work	4.04 (±2.75)
Leisure	1.13 (±1.61)
Home	0.29 (±0.54)
Money	1.22 (±1.43)
Religion	0.10 (±0.27)
Death	0.11 (±0.28)
**Physical**	2.26 (±1.96)
Health	0.88 (±1.18)
Sexual	0.03 (±0.14)
Food	0.89 (±0.79)
Death	0.11 (±0.28)
**Motives**	
Rewards	2.55 (±1.31)
Risk	2.18 (±0.99)
**Conversational**	0.26 (±0.37)
Netspeak	0.02 (±0.08)
Assent	0.16 (±0.28)
Non-fluencies	0.02 (±0.07)
Fillers	0.07 (±0.14)
**All punctuation**	13.85 (±2.65)
Periods	3.76 (±0.93)
Comma	7.43 (±2.21)
Question mark	0.12 (±0.40)
Exclamation points	0.02 (±0.20)
Apostrophes	0.05 (±0.24)
Other punctuation	2.39 (±1.69)

### Participants

As it can be seen in Table 1 presents detailed summaries of mood, sociodemographic and LIWC features of our sample of 62 college applicants.

Based on established clinical cutoffs for the PHQ-9 (Kroenke et al., 2001) and GAD-7 (Spitzer et al., 2006), 41 participants (66%) scored 10 or higher on the PHQ-9, indicating at least moderate depressive symptoms, while 34 participants (55%) scored 10 or higher on the GAD-7, indicating at least moderate anxiety symptoms. These proportions suggest a substantial presence of emotional distress among college applicants, consistent with literature on high-stress academic transitions.

### Generalized anxiety disorder 7-item (GAD-7)

Our objective was to investigate the sociodemographic and LIWC-related variables associated with each mood scale. So, as described in the data analysis topic, first, univariate regression analyzes were performed for all sociodemographic and LIWC variables, as independent variables, and the GAD-7 scores as dependent variable, in order to select the independent variables that would enter the first version of the multivariate model (theoretical relevance and variables that had a *p*-value < 0.2). Table 2 shows the final model for GAD-7, demonstrating that the main associated variables were: (i) Affiliation; (ii) Home; (iii) Leisure; and (iv) Money, are responsible for an adjusted R^2^ of 32.49%. The overall model fit was statistically significant, F(4, 57) = 8.34, *p* < 0.001, confirming the robustness of the associations identified. It is worth mentioning that, to control the model for any confounder, we test by age and sex. Lastly, regarding the four regression assumptions tested, the model met the requirements (same procedure was applied for PHQ-9 below).

**TABLE 2:** Multivariate regression analysis for generalized anxiety disorder 7-item (GAD-7).

Generalized anxiety disorder 7-item (GAD-7)	β	95% CI	*P*	R^2^
				32.49
Affiliation	1.01	0.36 to 1.64	0.003	–
Home	2.35	0.33 to 4.36	0.023	–
Leisure	−1.37	−2.08 to −0.66	<0.001	–
Money	−0.94	−1.74 to −0.15	0.020	–

### Patient Health Questionnaire-9 (PHQ-9)

Following the GAD-7 model, univariate regression analyzes were performed for all sociodemographic and LIWC variables, as independent variables, and the PHQ-9 scores as dependent variables, in order to select the independent variables that would enter the first version of the multivariate model. Table 3 shows the final model for PHQ-9, demonstrating that the main predictors were: (i) Drives; (ii) Motion; and (iii) Number, are responsible for an adjusted R^2^ of 18.15%. The overall model fit was statistically significant, F(3, 58) = 5.51, *p* = 0.002, supporting the adequacy of the selected predictors.

**TABLE 3:** Multivariate regression analysis for Patient Health Questionnaire-9 (PHQ-9).

Patient Health Question-naire-9 (PHQ-9)	β	95% CI	*P*	R^2^
				18.15
Drives	0.54	0.12 to 0.95	0.013	**–**
Motion	−1.17	−2.07 to −0.26	0.012	–
Number	1.52	0.19 to 2.85	0.026	**–**

## Discussion

This study aimed to determine whether the sampled population of college applicants exhibits mood impairments and to identify the linguistic associations with anxiety and depression levels. Our findings indicate that a significant portion of the evaluated sample demonstrated notable anxiety and depression levels. Specifically, 60% of the sample showed significant anxiety impairments. Multivariate regression models identified distinct sets of linguistic features associated with each outcome: for GAD-7, associations included affiliation, home, leisure, and money; for PHQ-9, associations included drives, motion, and number.

Although the sample displayed a high proportion of participants scoring above clinical cutoffs for depression and anxiety, the study’s design focused on capturing the dimensional relationship between linguistic features and emotional symptoms. This approach reflects the growing perspective in mental health research that symptom severity exists along a continuum, where linguistic markers may provide early indicators of emotional distress even below diagnostic thresholds. Thus, while not intended for categorical diagnosis, the findings reinforce the potential of text-based linguistic analysis as a low-cost, scalable screening tool for detecting early risk patterns in non-clinical populations.

Importantly, the current study was not designed to perform categorical discrimination between participants with and without clinically relevant symptomatology. Instead, the analyses focused on identifying linguistic features that co-varied with continuous measures of anxiety and depression. This dimensional approach is consistent with modern frameworks, such as the NIMH Research Domain Criteria (RDoC), which emphasize that psychological symptoms occur along a continuum rather than as discrete categories.

From a translational perspective, this finding suggests that linguistic markers derived from naturalistic writing may serve as early indicators of emotional distress, complementing traditional screening measures. While future research using clinical samples could explore diagnostic discrimination directly, our results provide preliminary evidence for the sensitivity of linguistic features to emotional and cognitive states associated with subclinical or moderate symptom levels.

Below, we further discuss the associative models for each dependent variable, exploring how these linguistic features are linked to anxiety and depression levels in pre-university students.

### Anxiety levels

Our analysis identified four main linguistic associations with anxiety levels among college applicants: affiliation, home, leisure, and money. These associations reflect the complex interplay between emotional states and language use, as evidenced by previous research in the field (Tausczik and Pennebaker, 2010; Ireland and Mehl, 2014; Tackman et al., 2019; Al-Mosaiwi and Johnstone, 2018).

Prior work has shown that linguistic markers — such as pronoun use, emotional vocabulary, and social or cognitive word categories — can serve as reliable indicators of underlying affective and cognitive states, both in experimental and real-world settings.

The use of words related to affiliation was positively associated with higher anxiety levels. This suggests that individuals with greater anxiety may have an increased need for social validation and support, possibly due to a heightened sense of insecurity or fear of rejection. This finding aligns with the broader literature, which highlights the role of social relationships in mental health. For instance, Lee and Robbins (1998) found that social connectedness is inversely related to anxiety and depression, suggesting that individuals who feel more connected to others experience lower levels of these symptoms. Furthermore, anxious individuals may use more affiliation-related language as a coping mechanism to seek reassurance from their social environment, as noted by Pennebaker and Seagal (2003) in their studies on expressive writing and emotional disclosure.

Words related to “home” and domestic life were also significantly associated with anxiety. This can be interpreted as a reflection of the desire for safety and stability that is often disrupted in anxious individuals. The home environment is typically associated with comfort and security, which may become focal points for those experiencing anxiety as they seek refuge from external stressors. Research by Taylor and Stanton (2008) supports this notion, indicating that individuals with high anxiety levels often exhibit a preference for familiar and controlled environments as a means of managing their symptoms.

A negative association was observed between anxiety levels and the use of words related to leisure activities. Higher anxiety levels were associated with fewer mentions of hobbies, sports, and other recreational activities. This finding is consistent with the concept of behavioral inhibition, where anxiety leads to a reduction in activities that are typically enjoyable and stress-relieving. This behavioral pattern is well-documented in the literature. Kashdan and Steger (2006) noted that individuals with anxiety disorders often withdraw from pleasurable activities, which in turn exacerbates their symptoms by reducing opportunities for positive reinforcement and social interaction. This withdrawal can create a vicious cycle, where decreased engagement in leisure activities leads to increased anxiety and further withdrawal.

Interestingly, fewer mentions of money-related terms were found among individuals with higher anxiety levels. One possible interpretation is that topics related to money and finances may be universally stress-inducing, and thus avoided by anxious individuals in their writing. Alternatively, the intense focus on academic performance and entrance exams may overshadow financial concerns, which are momentarily deprioritized. Research by Conger et al. (1999) suggests that financial stress can significantly impact mental health, leading to increased anxiety and depression. However, in the context of our study, the overwhelming stress associated with preparing for university entrance exams might eclipse financial anxieties, thus explaining the reduced mention of money-related terms.

Our findings are consistent with and expand upon existing research on the linguistic associations with mental health states. For example, Stirman and Pennebaker (2001) demonstrated that the use of certain linguistic categories, such as personal pronouns and emotion words, can predict psychological wellbeing. Similarly, our study underscores the importance of specific word categories in understanding anxiety levels among pre-university students.

### Depression levels

Our analysis identified three main linguistic associations with depression levels among college applicants: drives, motion, and number. These associations reflect the nuanced relationship between depressive symptoms and language use, consistent with previous research in the field.

Words related to drives, encompassing the internal force of life and psychological processes, were positively associated with higher levels of depressive symptoms. This suggests that students experiencing higher depression levels might be engaging in a deeper introspection, questioning the meaning and purpose of their efforts during the rigorous pre-university process. This aligns with findings by Chung and Pennebaker (2011), who noted that individuals with depressive tendencies often exhibit a heightened focus on internal states and existential questions in their language use. Such introspection, while a hallmark of depressive cognition, can also reflect an underlying struggle to find meaning in challenging circumstances.

The use of words related to motion was negatively associated with depression levels. Students with higher depression levels tended to use fewer words indicating movement. This lack of motion-related language may be indicative of the apathy and decreased initiative commonly seen in depressive disorders. Studies by Demyttenaere et al. (2005) support this, showing that psychomotor retardation and a general decrease in physical activity are prevalent symptoms of depression. The reduced mention of movement-related terms in the essays likely reflects this broader pattern of diminished engagement with the physical aspects of life, further underscoring the profound impact of depression on daily functioning.

Interestingly, words related to numbers were used more frequently by students with higher depression levels. This could suggest a focus on abstract and quantitative aspects rather than concrete experiences, as depressive individuals might distance themselves from direct, emotional, and sensory experiences. Research by Rude et al. (2004) found similar patterns, where individuals with depression tended to use more words associated with cognitive processes and less with sensory and perceptual experiences. This shift towards a more detached and analytical language style may reflect an attempt to impose structure and predictability in a context where emotional turmoil is prevalent.

These associations are consistent with and build upon existing literature on linguistic indicators of mental health states. For instance, Stirman and Pennebaker (2001) demonstrated that the use of certain linguistic categories, such as personal pronouns and emotion words, can predict psychological wellbeing. Similarly, our study highlights the importance of specific word categories in understanding depression levels among pre-university students.

### Clinical implications

The clinical implications of our study’s findings are significant, offering several potential applications for mental health assessment and intervention. Identifying symptoms of depression and anxiety through the analysis of written texts can enhance diagnostic accuracy, particularly in challenging cases where traditional diagnostic methods may fall short. This approach leverages the functional aspects of language to reveal underlying psychological states, thereby providing a more comprehensive picture of an individual’s mental health. In clinical settings, the ability to detect depressive and anxious symptoms in youths’ written texts could facilitate early intervention. By integrating linguistic analysis into routine mental health assessments, clinicians can identify at-risk individuals more effectively. This method can serve as a supplementary tool to traditional assessments, offering insights that might not be captured through standard questionnaires and interviews. The enhanced diagnostic accuracy can lead to more tailored and timely interventions, ultimately improving patient outcomes.

Preliminary screening of patients using linguistic analysis of formal texts, such as work or school assignments, can also aid in the early detection of mental health issues. By analyzing these texts, healthcare providers can identify subtle signs of depression and anxiety that might otherwise go unnoticed. This proactive approach allows for earlier and potentially more effective intervention strategies, reducing the risk of symptom escalation and associated complications.

In educational settings, the implications are equally profound. School administrators and counselors can use linguistic analysis to identify students who exhibit signs of anxiety and depression. By monitoring written assignments, educators can flag students who may need additional support, prompting further investigation and appropriate interventions. This can help create a more supportive school environment, where mental health issues are addressed promptly and effectively, thereby fostering better academic and personal development among students.

Furthermore, the use of linguistic analysis in workplace settings can enhance employee well-being. By incorporating this method into routine employee assessments, organizations can identify individuals who may be struggling with anxiety or depression levels. Early detection allows for timely interventions, such as counseling or stress management programs, which can improve employee productivity and overall workplace morale. This approach not only benefits the individual employees but also contributes to a healthier, more supportive work environment. Overall, the application of linguistic analysis for identifying anxiety and depression levels in various contexts represents a promising avenue for improving mental health care. By utilizing this innovative approach, clinicians, educators, and employers can better understand and address the mental health needs of those under their care, leading to more effective and holistic interventions.

### Public health implications

The public health implications of our study are far-reaching, providing valuable insights into the prevalence and impact of depressive and anxious symptoms among pre-university students. By utilizing linguistic analysis as a tool to assess mental health through written texts, even formal ones, we can enhance demographic analyses of these symptoms. This approach offers a novel means for government bodies and public health officials to understand the mental health landscape more comprehensively and to develop targeted strategies for awareness, prevention, and treatment of depression and anxiety levels.

Implementing this tool in pre-university courses and schools allows for a deeper exploration of the context in which students exhibit depressive and anxious symptoms. By analyzing written assignments, educators and policymakers can gain a clearer picture of the mental health challenges faced by students. This, in turn, enables the formulation of more specific and effective public policies aimed at supporting student mental health. Such targeted interventions can help mitigate the negative effects of anxiety and depression levels, promoting better educational outcomes and overall wellbeing.

Moreover, the findings of this study call into question the current methods of student evaluation, which predominantly rely on written exams and multiple-choice tests. Given that mental health issues can significantly influence students’ performance on these assessments, it is crucial to consider whether these methods fairly evaluate students’ abilities. The stress and pressure associated with high-stakes testing can exacerbate mental health problems, particularly among vulnerable populations. This insight suggests a need to reevaluate and potentially reform the rules and regulations governing university entrance exams, as established by bodies such as the Ministry of Education. By incorporating alternative assessment methods that recognize diverse intelligences and reduce undue stress, educational systems can create a more equitable and supportive environment for all students.

Furthermore, the insights gained from linguistic analysis of student texts can inform broader public health initiatives. By identifying demographic patterns and high-risk groups, public health officials can design and implement programs that address the specific needs of different communities. For example, targeted mental health campaigns can be developed to raise awareness about the signs and symptoms of depression and anxiety, encouraging early intervention and reducing stigma. Prevention programs can be tailored to the unique challenges faced by pre-university students, providing them with the tools and resources needed to manage stress and maintain mental health.

### Limitations

This study has several limitations that should be acknowledged. First, the sample size was relatively small and limited to students from pre-university courses in São Paulo, which may not be representative of the broader population of college applicants. This limits the generalizability of our findings to other contexts and regions.

Second, the study relied on self-reported data for the PHQ-9 and GAD-7 scales, which may be subject to response biases such as social desirability or recall bias. Additionally, while the use of LIWC for linguistic analysis provides valuable insights, it is limited to the predefined categories within the software and may not capture the full complexity of language use related to mental health.

Third, the cross-sectional design of the study precludes the establishment of causal relationships between linguistic features and mental health outcomes. Longitudinal studies would be necessary to determine whether changes in language use over time correlate with changes in anxiety and depression levels.

Finally, the study did not account for other potential confounding variables, such as socioeconomic status, previous mental health history, or other environmental factors, which could influence both language use and mental health outcomes. Future research should consider these variables to provide a more comprehensive understanding of the associations observed.

## Conclusion

This study underscores the potential of linguistic analysis as a valuable tool for identifying and understanding the psychological states of pre-university students, revealing significant associations between specific language use and levels of anxiety and depression. By highlighting these associations, our findings contribute to the growing body of evidence that written language can serve as a window into mental health, offering novel avenues for early detection and intervention. Implementing such methods in clinical, educational, and public health settings could enhance the accuracy of mental health assessments, inform more targeted and effective interventions, and ultimately improve the wellbeing and academic performance of students. Future research should continue to explore the dynamic interplay between language and mental health, leveraging advanced linguistic technologies to further refine these predictive models and extend their applicability across diverse populations and contexts.

## Data Availability

The raw data supporting the conclusions of this article will be made available by the authors, without undue reservation.

## References

[B1] Al-Mosaiwi M. Johnstone T. (2018). In an absolute state: Elevated use of absolutist words is a marker of mental health disorders. *Clin. Psychol. Sci.* 6 529–546. 10.1177/2167702617747074 30886766 PMC6376956

[B2] Balage Filho P. P. Pardo T. A. S. Aluísio S. M. (2013). “An evaluation of the Brazilian Portuguese LIWC dictionary for sentiment analysis,” in *Proceedings of the 9th Brazilian symposium in information and human language technology*, (Porto Alegre: SBC).

[B3] Bursac Z. Gauss C. H. Williams D. K. Hosmer D. W. (2008). Purposeful selection of variables in logistic regression. *Source Code Biol. Med.* 3:17. 10.1186/1751-0473-3-17 19087314 PMC2633005

[B4] Chung C. Pennebaker J. (2011). “*The psychological functions of function words*,” in Social communication (Psychology Press), 343–359.

[B5] Conger R. D. Conger K. J. Elder G. H. (1999). “Family economic hardship and adolescent adjustment: Mediating and moderating processes,” in *Economic stress: New directions in research on mental health and poverty*, eds Bickel W. J. Dawson C. W. (Washington, DC: American Psychological Association), 59–87.

[B6] de Barbosa F. O. Macedo P. C. M. Silveira R. M. C. (2011). Depressão e o suicídio. [Depression and suicide]. *Rev. SBPH* 14 233–243. 10.57167/Rev-SBPH.14.401 Portuguese

[B7] Demyttenaere K. De Fruyt J. Stahl S. M. (2005). The many faces of fatigue in major depressive disorder. *Int. J. Neuropsychopharmacol*. 8, 93–105. 15482632 10.1017/S1461145704004729

[B8] Ireland M. E. Mehl M. R. (2014). Natural language use as a marker of personality. *Curr. Direct. Psychol. Sci.* 23 319–323. 10.1037/pspp0000187 29504797

[B9] Kashdan T. B. Steger M. F. (2006). Expanding the topography of social anxiety: An experience-sampling assessment of positive emotions, positive events, and emotion suppression. *Psychol. Sci.* 17 120–128. 10.1111/j.1467-9280.2006.01674.x 16466419

[B10] Kroenke K. Spitzer R. L. Williams J. B. W. (2001). The PHQ-9: Validity of a brief depression severity measure. *J. General Intern. Med.* 16 606–613. 10.1046/j.1525-1497.2001.016009606.x 11556941 PMC1495268

[B11] Lee R. M. Robbins S. B. (1998). The relationship between social connectedness and anxiety, self-esteem, and social identity. *J. Counsel. Psychol.* 45 338–345. 10.1037/0022-0167.45.3.338

[B12] Osborne J. W. Waters E. (2002). Four assumptions of multiple regression that researchers should always test. *Pract. Assess. Res. Eval.* 8:2. 10.7275/r222-hv23

[B13] Pennebaker J. W. (2001). *Linguistic inquiry and word count*.

[B14] Pennebaker J. W. Seagal J. D. (2003). Forming a story: The health benefits of narrative. *J. Clin. Psychol.* 55 1243–1254. 10.1002/jclp.10126 11045774

[B15] Rude S. S. Gortner E. M. Pennebaker J. W. (2004). Language use of depressed and depression-vulnerable college students. *Cogn. Emot.* 18 1121–1133. 10.1080/02699930441000030

[B16] Spitzer R. L. Kroenke K. Williams J. B. W. Löwe B. (2006). A brief measure for assessing generalized anxiety disorder: The GAD-7. *Arch. Intern. Med.* 166 1092–1097. 10.1001/archinte.166.10.1092 16717171

[B17] Stirman S. W. Pennebaker J. W. (2001). Word use in the poetry of suicidal and nonsuicidal poets. *Psychosomatic Med.* 63 517–522. 10.1097/00006842-200107000-00001 11485104

[B18] Tackman A. M. Sbarra D. A. Carey A. L. Donnellan M. B. Horn A. B. Holtzman N. S. (2019). The psychological significance of subtle linguistic markers: A meta-analysis. *Psychol. Bull.* 145 1173–1199. 10.1037/pspp0000187 29504797

[B19] Tausczik Y. R. Pennebaker J. W. (2010). The psychological meaning of words: LIWC and computerized text analysis methods. *J. Lang. Soc. Psychol.* 29 24–54. 10.1177/0261927X09351676

[B20] Taylor S. E. Stanton A. L. (2008). Coping resources, coping processes, and mental health. *Ann. Rev. Clin. Psychol.* 1 377–401. 10.1146/annurev.clinpsy.1.102803.144141 17716061

[B21] Terra D. H. P. Vieira G. A. Costa A. M. D. D. Terra F. D. S. Freire G. E. R. (2013). Ansiedade e depressão em vestibulandos. [Anxiety and depression in college entrance exam candidates]. *Odontol. Clínico-Científ.* 12 273–276. Portuguese

[B22] Walker A. Zirikly A. Stockbridge M. Wilcox H. C. (2024). A linguistic analysis of instagram captions between adolescent suicide decedents and living controls. *Crisis* 45 136–143. 10.1027/0227-5910/a000928 37818627

[B23] World Health Organization [WHO] (2020). *Depression and other common mental disorders: Global health estimates.* Geneva: WHO.

